# Photobiomodulation therapy in management of cancer therapy-induced side effects: WALT position paper 2022

**DOI:** 10.3389/fonc.2022.927685

**Published:** 2022-08-30

**Authors:** Jolien Robijns, Raj G. Nair, Joy Lodewijckx, Praveen Arany, Andrei Barasch, Jan M. Bjordal, Paolo Bossi, Anne Chilles, Patricia M. Corby, Joel B. Epstein, Sharon Elad, Reza Fekrazad, Eduardo Rodrigues Fregnani, Marie-Thérèse Genot, Ana M. C. Ibarra, Michael R. Hamblin, Vladimir Heiskanen, Ken Hu, Jean Klastersky, Rajesh Lalla, Sofia Latifian, Arun Maiya, Jeroen Mebis, Cesar A. Migliorati, Dan M. J. Milstein, Barbara Murphy, Judith E. Raber-Durlacher, Hendrik J. Roseboom, Stephen Sonis, Nathaniel Treister, Yehuda Zadik, René-Jean Bensadoun

**Affiliations:** ^1^ UHasselt, Faculty of Medicine and Life Sciences, Diepenbeek, Belgium; ^2^ Oral Medicine, Oral Pathology and Oral Oncology, Griffith University, Department of Haematology and Oncology, Gold Coast University Hospital, Gold Coast, QL, Australia; ^3^ School of Dental Medicine, Oral Biology and Biomedical Engineering, University at Buffalo, Buffalo, NY, United States; ^4^ Harvard School of Dental Medicine, Division of Oral Medicine and Dentistry, Boston, MA, United States; ^5^ Physiotherapy Research Group, IGS, University of Bergen, Bergen, Norway; ^6^ Department of Medical and Surgical Specialties, Radiological Sciences and Public Health, University of Brescia, Brescia, Italy; ^7^ Radiotherapy Department, Institut Curie, Paris, France; ^8^ New York University College of Dentistry, Bluestone Center for Clinical Research, New York, NY, United States; ^9^ City of Hope Duarte, CA and Cedars-Sinai Health System, Los Angeles, CA, United States; ^10^ Eastman Institute for Oral Health, University of Rochester Medical Center, Rochester, NY, United States; ^11^ Department of Periodontology, Dental Faculty – Radiation Sciences Research Center, Laser Research Center in Medical Sciences, AJA University of Medical Sciences, Tehran, Iran; ^12^ Oral Medicine Department, Hospital Sírio-Libanês, São Paulo, Brazil; ^13^ Laser Therapy Unit, Institut Jules Bordet, Centre des Tumeurs de l’Université Libre de Bruxelles, Brussels, Belgium; ^14^ Postgraduate Program on Biophotonics Applied to Health Sciences, Nove de Julho University, São Paulo, Brazil; ^15^ Wellman Center for Photomedicine, Massachusetts General Hospital, Harvard Medical School, Boston, MA, United States; ^16^ Oral and Maxillofacial Diseases, University of Helsinki, Helsinki, Finland; ^17^ Department of Radiation Oncology, NYU Langone Health, New York, NY, United States; ^18^ Medical Oncology, Institut Jules Bordet, Brussels, Belgium; ^19^ Section of Oral Medicine, University of Connecticut School of Dental Medicine, Farmington, CT, United States; ^20^ Department of Medicine, Institut Jules Bordet, Universiteí Libre de Bruxelles, Brussels, Belgium; ^21^ Manipal College of Health Professions, MAHE, Manipal, India; ^22^ Department of Oral and Maxillofacial Diagnostic Sciences, University of Florida College of Dentistry, Gainesville, Florida, United States; ^23^ Oral and Maxillofacial Surgery, Academic Medical Center, University of Amsterdam, Amsterdam, Netherlands; ^24^ Department of Oncology, Vanderbilt University Medical Center, Nashville, TN, United States; ^25^ Department of Oral and Maxillofacial Surgery, Amsterdam UMC, University of Amsterdam, Department of Oral Medicine, Academic Centre for Dentistry Amsterdam, University of Amsterdam and VU University, Amsterdam, Netherlands; ^26^ Division of Oral Medicine and Dentistry, Brigham and Women’s Hospital; Department of Oral Medicine, Infection and Immunity, Harvard School of Dental Medicine; Division of Oral Medicine and Dentistry, Brigham and Women’s Hospital, Dana-Farber Cancer Institute, Boston, MA, United States; ^27^ Department of Military Medicine, Faculty of Medicine, Hebrew University of Jerusalem, Jerusalem, Israel, and Department of Oral Medicine, Sedation and Maxillofacial Imaging, Hebrew University-Hadassah School of Dental Medicine, Jerusalem, Israel; ^28^ Department of Radiation Oncology, Centre de Haute Energie, Nice, France

**Keywords:** photobiomodulation (PBM), cancer supportive care, guidelines, recommendations, mucositis, dermatitis, cancer-treatment side effects

## Abstract

**Disclaimer:**

This article is based on recommendations from the 12^th^ WALT Congress, Nice, October 3-6, 2018, and a follow-up review of the existing data and the clinical observations of an international multidisciplinary panel of clinicians and researchers with expertise in the area of supportive care in cancer and/or PBM clinical application and dosimetry. This article is informational in nature. As with all clinical materials, this paper should be used with a clear understanding that continued research and practice could result in new insights and recommendations. The review reflects the collective opinion and, as such, does not necessarily represent the opinion of any individual author. In no event shall the authors be liable for any decision made or action taken in reliance on the proposed protocols.

**Objective:**

This position paper reviews the potential prophylactic and therapeutic effects of photobiomodulation (PBM) on side effects of cancer therapy, including chemotherapy (CT), radiation therapy (RT), and hematopoietic stem cell transplantation (HSCT).

**Background:**

There is a considerable body of evidence supporting the efficacy of PBM for preventing oral mucositis (OM) in patients undergoing RT for head and neck cancer (HNC), CT, or HSCT. This could enhance patients’ quality of life, adherence to the prescribed cancer therapy, and treatment outcomes while reducing the cost of cancer care.

**Methods:**

A literature review on PBM effectiveness and dosimetry considerations for managing certain complications of cancer therapy were conducted. A systematic review was conducted when numerous randomized controlled trials were available. Results were presented and discussed at an international consensus meeting at the World Association of photobiomoduLation Therapy (WALT) meeting in 2018 that included world expert oncologists, radiation oncologists, oral oncologists, and oral medicine professionals, physicists, engineers, and oncology researchers. The potential mechanism of action of PBM and evidence of PBM efficacy through reported outcomes for individual indications were assessed.

**Results:**

There is a large body of evidence demonstrating the efficacy of PBM for preventing OM in certain cancer patient populations, as recently outlined by the Multinational Association for Supportive Care in Cancer/International Society of Oral Oncology (MASCC/ISOO). Building on these, the WALT group outlines evidence and prescribed PBM treatment parameters for prophylactic and therapeutic use in supportive care for radiodermatitis, dysphagia, xerostomia, dysgeusia, trismus, mucosal and bone necrosis, lymphedema, hand-foot syndrome, alopecia, oral and dermatologic chronic graft-versus-host disease, voice/speech alterations, peripheral neuropathy, and late fibrosis amongst cancer survivors.

**Conclusions:**

There is robust evidence for using PBM to prevent and treat a broad range of complications in cancer care. Specific clinical practice guidelines or evidence-based expert consensus recommendations are provided. These recommendations are aimed at improving the clinical utilization of PBM therapy in supportive cancer care and promoting research in this field. It is anticipated these guidelines will be revised periodically.

## 1 Introduction

Despite the ongoing improvements in cancer therapy, it is still associated with severe life-impairing side effects. Both treatment- and patient-related risk factors determine the severity of the complications. Moreover, they negatively impact the patients’ quality of life (QoL) and daily activities. Therefore, effective supportive care strategies are necessary (1). The biological effects of photobiomodulation (PBM) therapy were discovered by Endre Mester in 1965 (2). Currently, PBM is defined as: “the use of non-ionizing optical radiation in the visible and near-infrared spectral range is absorbed by endogenous chromophores to elicit photophysical and photochemical events at various biological scales without eliciting thermal damage, leading to physiological changes and therapeutic benefits” (3). There is a considerable body of evidence supporting the efficacy of PBM for the prevention of oral mucositis (OM) in patients undergoing radiotherapy (RT) for head and neck cancer (HNC), chemotherapy (CT), or hematopoietic stem cell transplantation (HSCT) (4). Recent advances in understanding the mechanisms of action of PBM and dosimetry parameters of PBM have resulted in examining other oncology-related conditions that may lead to effective management of a broader range of complications associated with cancer treatment. This could improve overall QoL, adherence to cancer treatment regimens, and their outcomes while reducing cost of care (5).

## 2 Methods

This position paper was developed based on reviewing existing literature on PBM in supportive cancer care. For oral mucositis, a systematic review was feasible. For all other indications, expert consensus opinions are based on a narrative review of the literature and the personal experiences of contributing experts. A level of evidence was assigned to these indications based on the Somerfield criteria based on the type and design of the study (6). The level of evidence for each intervention is then translated into a guideline recommendation where Level I or II evidence is achieved by at least one well-designed randomized controlled trial (RCT). A suggestion was possible for lower-level evidence, but only when consistent evidence from multiple studies and panel consensus on the interpretation of this evidence. Thus, to enable maximal, practical clinical use and promote future research for PBM in supportive cancer care, WALT makes recommendations in two categories as clinical practice guidelines and consensus expert opinion. We strongly recommend clinical judgment of the provider and individual patient scenario be carefully éaccounted for in interpreting and applying these WALT PBM recommendations.

## 3 Results

The reader is referred to comprehensive reviews in these WALT series for a detailed discussion of PBM parameters. A brief review of critical PBM parameters is presented below.

### 3.1 PBM parameters

PBM parameters using low-level lasers or light-emitting diodes (LEDs) in cancer supportive care (summarized in **Table 1**) are usually within the red and near-infrared (NIR) wavelength range between 600 nanometers (nm) and around 1,000 nm, with a power density from 5 (mW)/cm^2^ to 150 mW/cm^2^ (7). The duration of application varies according to the site, but it may well be within 30 - 60 seconds per point. While shorter efficacious treatment times have been used (2-10sec per point, multiple spots that are clinically laborious), this could be attributed to cumulative PBM dose effects. The therapeutic dosage is depicted as the energy density measured in Joules as J/cm^2^ and varies between 0.1 to 12 J/cm^2^ as per current literature. Low-level laser systems used include helium-neon (HeNe), neodymium-doped yttrium aluminum garnet (Nd : YAG), gallium aluminum arsenide (GaAlAs) diode lasers, indium gallium aluminum phosphorus (InGaAlP), and non-thermal, non-ablative carbon dioxide (CO_2_) lasers (8). In recent years, LEDs with wavelengths in the red or NIR regions have become increasingly common due to their safety, low cost, and suitability for home use (8, 9).

**Table 1:** WALT 2022 recommendations for PBM in the management of OM – updated MASCC/ISSO guidelines from Zadik et al., 2019 (4).

Cancer Treatment Modality	Treatment Intent	PBM Treatment Parameters										
		Device Parameters							Delivery parameters			
		Wavelength(nm)	Irradiance (Power Density) (mW/cm2)	Time per spot(sec)	Fluence (Energy Density) (J/cm2)	Photon Fluence (p.J/cm2)	Einstein(E)	Spot size(cm2)	Number of spots	Distance from the tissue	Frequency	Duration
HSCT	Prevention	632.8	31.25	40	1.0	2	0.4	0.8	18	< 1 cm	Daily	From day after cessation of conditioning for 5 days
		650	1000 *	2	2.0	3.8	0.8	0.04	54-70	In contact	Daily	From 1st day of conditioning till day + 2 post-HSCT (for 7-13 days)
RT	Prevention	632.8	24	125	3.0	6	1.3	1	12	< 1 cm	5 days/wk	Entire RT course
RT-CT	Prevention	660	417 *	10	4.2	8.0	1.7	0.24	72	In contact	5 days/wk	Entire RT course
		660	625 *	10	6.2	11.8	2.6	0.04	69	In contact	3 days/wk (alternate days)	Entire RT course

*Potential thermal effect; The clinician is advised to pay attention to the combination of specific parameters.

CT, chemotherapy; HSCT, hematopoietic stem cell transplantation; IO, intra-oral; NR, not reported; PBM, photobiomodulation; RT, radiotherapy; wk, week;

1 Einstein = 4.5 p.J/cm2 which is the photonic fluence at 810 nm that is equivalent to the conventional fluence of 3 J/cm2 (62).

Photon Energy at 632 nm = 2 eV and 650 and 660 nm = 1.9 eV.

**Table 2:** WALT 2022 recommendations for PBM in managing salivary gland dysfunction in cancer patients - updated from Heiskanen et al., 2020 (78).

Ref.	Patient population	Treatment Intent	Study design	PBM Treatment Parameters										Efficacy
				Route of delivery	Wavelength (nm)	Laser type	Approach	Power(mW)	Irradiance(mW/cm2)	Time(sec)	Fluence (J/cm2)	Photon Fluence (p.J/cm2)	Einstein(E)	
Gonnelli 2016 (108)	HNC	P	RCT	Combo	660 +780 + clinical care	InGalAlP	IO (660 nm)	40	Calculated:1000	10	10	19	4.2	Yes, objective outcome measures
							EO (780 nm)	15	Calculated:375	10	3.8	5.7	1.3
Palma 2017 (110)	HNC	T	Before-and-after	Combo	808	InGalAlP	IO	30	750	10	7.5	11.2	2.5	Yes, objective and subjective outcome measures
							EO						
Saleh 2014 (111)	HNC	T	RCT	Combo	830	GaAlAr	IO	100	3570	20	71	106.5	23.7	Not effective in both objective and subjective outcome measures
				EO									
Simoes 2010 (112)	HNC	P	Comparative	Intraoral	660	AlGaInP	IO	40	Calculated:1111	6	6	11.4	2.5	Yes, subjective outcome measures
Cowen 1997 (71)	Hematologic cancer	P	RCT	Intraoral	632.8	HeNe	IO	60	Calculated:150	10	1.5	3	0.7	Yes, subjective outcome measures

intra-oral; EO, extra-oral; HNC head and neck cancer; HSCT, hematopoietic stem cell transplantation; P, prevention; Ref, reference; RCT, randomized controlled trial; T, therapy

1 Einstein = 4.5 p.J/cm2 which is the photonic fluence at 810 nm that is equivalent to the conventional fluence of 3 J/cm2 (62).

Photon Energy at 632 nm = 2 eV and 660 nm = 1.9 eV, 780 = eV, 808 = 1.5 eV, and 830 nm = 1.5eV.

**Table 3:** WALT 2022 recommendations for PBM treatments in prevention and/or management of cancer therapy-related complications.

Complication	PBM Treatment Parameters												Level of Evidence*I to V	Level of Evidence Recommendation or Expert Opinion
	Device parameters									Delivery parameters				
	Route of delivery	Beam Mode(Continuous and/or Pulsed)	Wavelength(nm)	Power(mW)	Irradiance (mW/cm²)	Time(sec)	Specified at 810 nm			Treatment area	Distance from tissue(Contact/non-contact)	Frequency(No. sessions/week andTotal sessions)		
							Fluence(J/cm²)(Prophylactic or Curative intent)	Photon Fluence (p.J/cm2)	Einstein(E)					
Acute Radiodermatitis	External	CW &/P	630-904	20-150	20-150	TBD	3	4.5	1	TBD	TBD	Daily10->30	II	Expert opinion
							6	9	2
Lymphedema	External	CW &/P	750-904	20-150	20-150 (Red)20-80 (IR)	TBD	2	3	0.7	TBD	TBD	3 times a week for 4 - 6 weeks	III	Expert Opinion
							6	9	2
Radiation Fibrosis	External & Internal	CW &/P	750-850	20-150	20-150 (Red)20-80 (IR)	TBD	2	3	0.7	TBD	TBD	3 times a week for 4 - 6 weeks	NA	Expert Opinion
							6	9	2
Palmar-Plantar Erythrodysesthesia	External	CW &/P	630-680+750-850	20-150	20-150 (Red)20-80 (IR)	TBD	2	3	0.7	TBD	TBD	3 times a week for 4 - 6 weeks	V	Expert Opinion
							6	9	2
Graft versus Host disease	External & Internal	CW &/P	630-680+750-850	20-150	20-150 (Red)20-80 (IR)	TBD	2	3	0.7	TBD	TBD	3 times a week for 4 - 6 weeks	IV	Expert Opinion
							6	9	2
Dysphagia	External & Internal	CW &/P	630-680+750-850	20-150	20-150 (Red)20-80 (IR)	TBD	2	3	0.7	TBD	TBD	3 times a week for 4 - 6 weeks	V	Expert Opinion
							6	9	2
Dysgeusia	External & Internal	CW &/P	630-680+750-850	20-150	20-150 (Red)20-80 (IR)	TBD	2	3	0.7	TBD	TBD	3 times a week for 4 - 6 weeks	V	Expert Opinion
							6	9	2
Trismus	External & Internal	CW &/P	630-680+750-850	20-150	20-150 (Red)20-80 (IR)	TBD	2	3	0.7	TBD	TBD	3 times a week for 4 - 6 weeks	V	Expert opinion
							6	9	2
Osteonecrosis and Mucosal necrosis	External & Internal	CW &/P	630-680+750-850	20-150	20-150 (Red)20-80 (IR)	TBD	2	3	0.7	TBD	TBD	3 times a week for 4 - 6 weeks	IV	Expert Opinion
							6	9	2
Voice and/or Speech alterations	External	CW &/P	630-680+750-850	20-150	20-150 (Red)20-80 (IR)	TBD	2	3	0.7	TBD	TBD	3 times a week for 4 - 6 weeks	NA	Expert Opinion
							6	9	2
Chemotherapy-Induced Peripheral Neuropathy	External	CW &/P	780-970	80-120	20-150 (Red)20-80 (IR)	TBD	7.5	11.2	2.5	TBD	TBD	3 times a week for 4 - 6 weeks	IV	Expert Opinion
							48	72	16
Chemotherapy-Induced Alopecia	External	CW &/P	630-680+750-850	20-150	20-150 (Red)20-80 (IR)	TBD	2	3	0.7	TBD	TBD	3 times a week for 4 - 6 weeks	NA	Expert Opinion
							6	9	2
Periodontal lesions after Chemotherapy and Radiotherapy	Internal	CW &/P	630-660+810-830	20-150	20-150 (Red)20-80 (IR)	TBD	2	3	0.7	TBD	TBD	Daily10 to 30	NA	Expert Opinion
							6	9	2

NA, not applicable TBD: to be decided by the operator

These proposed protocols are based on expert opinion and do not exclude other protocols. *Level I: Evidence obtained from meta-analysis of multiple, well-designed, controlled studies; randomized trials with low false-positive and false-negative errors (high power); Level II: Evidence obtained from at least 1 well-designed experimental study; randomized trials with high false-positive and/or false-negative errors (low power); Level III: Evidence obtained from well-designed, quasi-experimental studies such as nonrandomized, controlled single-group, pre-test, and post-test comparison, cohort, time, or matched case-control series; Level IV: Evidence obtained from well-designed, non-experimental studies, such as comparative and correlational descriptive and case studies; Level V: Evidence obtained from case reports and clinical examples (48).

1 Einstein = 4.5 p.J/cm2 which is the photonic fluence at 810 nm that is equivalent to the conventional fluence of 3 J/cm2 (62).

Photon Energy at 632 nm = 2 eV, 660 nm = 1.9 eV, 680 nm = 1.8 eV, 750 nm = 1.6 eV, 780 = 1.5 eV, 808 = 1.5 eV, and 850 nm = 1.5eV, 904 nm = 1.4 eV.

The biological effects of PBM on the exposed tissues depend upon a number of variables, including the location of the cells in the field of exposure, cell type, molecular and redox state of the cell, the tissue microenvironment, PBM parameters such as wavelength, power density, type of delivery as in pulsing or continuous, beam or spot size, and duration of exposure (10, 11). It is well known that PBM therapy exhibits a biphasic dose-response that warrants optimal tissue-specific irradiation dose parameters. In other words, doses lower than the optimal value may have a diminished effect, while doses higher than optimal can have no beneficial or even adverse therapeutic outcomes (12, 13). The effect of such a phenomenon has been consistently evident in published data on the effectiveness and particular disparity of PBM therapy in cancer complications.

Titrating adequate doses and defining the essential PBM parameters as per evidence gathered in a systematic way for each indication is a prerequisite for the successful use of this treatment modality. Without standardization in beam measurement, dose calculation, and the correct reporting of these parameters, studies will not be reproducible, and outcomes will not be consistent. A common misconception is that energy (in J) or energy density (J/cm^2^) is all that is necessary to replicate a successful treatment, irrespective of the original power, power density, and duration parameters (14, 15). In addition, it is not uncommon to find discrepancies between the specifications provided by a device manufacturer and the actual performance of the device (16). Therefore, routine device maintenance, including power measurements, should be carried out regularly in the context of research trials and clinical practice.

### 3.2 Safety considerations

During the course of more than two decades of PBM use in the management of OM in HNC patients, limited significant adverse effects have been reported. Only one study reported a burning sensation following PBM therapy in 50% of pediatric patients (9 out 18) (17). Given its diverse biological impact, consideration of PBM on tumor response to therapy and/or tumor behaviors remains a critical question that has yet to be definitively answered. Given tumor genomic heterogeneity, it seems likely that the effect of PBM on tumor behavior, like drugs or biologicals, is not uniform and might provide an explanation that addresses the contradictions of observations reported in the literature. Even tumors of similar histological characterization (i.e., squamous cell cancers of the mouth) vary, as is illustrated by 35% expressing dysregulations in the PI3K pathway, a common PBM target (18). It is important to note the limitations of *in vitro* studies in oncology versus the systems approach and clinical outcomes that are required. Clinical trials focus more on the effects of PBM on epithelial and connective tissue interactions, micro-environment, immune recognition, and on immune function. While cell culture studies may provide some insight into potential mechanisms, *in vivo* and human trial data are mandatory, and no firm conclusions can be drawn using tissue culture studies alone.

#### 3.2.1 *In vitro* and *in vivo* safety data

It is unlikely that PBM has carcinogenic effects in normal cells. The non-ionizing wavelengths of the red and NIR spectrum used in PBM are far longer than the safety limit of 320 nm for DNA damage (19–21). No signs of malignant transformation in non-malignant epithelial cells and fibroblasts were observed following exposure to PBM with a wavelength of 660 nm, 350 mW for 15 minutes during three consecutive days (22). In addition, no malignant transformation of normal breast epithelial cells was detected in an *in vitro* study comparing the effects of different doses and wavelengths of PBM during multiple exposures (23).

Conflicting data refute or support the potential for PBM to impact tumor activity and responsiveness. As noted above, given the lack of uniformity, which characterizes tumor biology, it seems probable that tumors might vary widely in how they react to the range of biomodulatory activities that results from PBM exposure. The literature is rich with papers in this space. Many of the pathways associated with negative tumor behaviors are induced by PBM, including cell proliferation and anti-apoptosis. In fact, the effects of PBM on cell proliferation and differentiation have been investigated in cell culture systems *in vitro* using malignant cell lines, and have generated contradictory data across a range of different tumor cell lines and PBM parameters (24–29). For example, a study with laryngeal carcinoma cells demonstrated proliferation after 809 nm GaAIAs laser irradiation at energy densities between 1.96 and 7.84 J/cm^2^ (19). Another study also found increased cell proliferation of HEp2 carcinoma cells after PBM exposure at different wavelengths (685 nm and 830 nm) and doses (30). In a study comparing PBM administered to normal osteoblasts and osteosarcoma cells with a range of different wavelengths and doses, only 10 J/cm^2^ from an 830 nm laser was able to enhance osteoblast proliferation, whereas energy densities of 1 J/cm^2^, 5 J/cm^2^, and 10 J/cm^2^ from a 780 nm laser decreased proliferation. Osteosarcoma cells were unaffected by 830 nm laser irradiation, whereas 670 nm laser had a mild proliferative effect (31).

An *in vitro* study compared the effects of different doses of PBM at various wavelengths on human breast carcinoma and melanoma cell lines (23). Although certain doses of PBM increased breast carcinoma cell proliferation, multiple exposures had either no effect or showed negative dose-response relationships. PBM (660nm) administered in low doses (1 J/cm^2^) increased *in vitro* proliferation and potentially increased invasive potential of tongue squamous cell carcinoma cells (32). Similarly, another *in vitro* study suggested that PBM (660 nm or 780 nm, 40 mW, 2.05, 3.07 or 6.15 J/cm^2^) might stimulate oral dysplastic and oral cancer cells by modulating the Akt/mTOR/CyclinD1 signaling pathway to produce a more aggressive behavior (33). PBM exposure to three HNC cell lines was noted to result in the proliferation of cells in each tumor line, but not in a normal tissue control (34). A systematic review demonstrated that the effect of PBM on tumor cells depends highly on the PBM parameters used (35). While the limits of basing broad-reaching conclusions on *in vitro* assays have been noted, collectively, it would be irresponsible to ignore the possibility that PBM could, in some cases, negatively impact tumor behavior. Investigating and understanding how PBM may modify tumor behaviors, both positively and negatively, is a research priority.

Direct investigation of the radiation effects of PBM as it affects tumor response is limited, but as with other forms of cytotoxic cancer therapy, it is likely that PBM may have the ability to affect tumor response to radiation in ways, which are informed, not only by the dose, fractions, and timing of PBM or radiotherapy (RT) but by the tumor. While the data is sparse and limited to *in vitro* systems, there is evidence to suggest that, in some cases, PBM may act as a radiosensitizer (36). High fluences (120 J/cm^2^) have been noted to up-regulate the activity of survivin, a member of the inhibitor of apoptosis family (IAP), mediating self-protection during tumor cell apoptosis (37). An *in vitro* study observed a pro-apoptotic effect of PBM in oral squamous cell carcinoma (OSCC) cells in the absence of radiation and no anti-apoptotic effects occurred that might promote tumor cell resistance to cancer therapy (22). Increased apoptosis of human osteosarcoma cells was also induced by the administration of NIR (810 nm, continuous-wave, 20 mW/cm², 1.5 J/cm²) prior to NPe6-mediated photodynamic therapy as a result of increased cellular ATP and a higher uptake of the photosensitizer (38). On the potential enhancement of ionizing RT and CT, a study demonstrated that PBM applied shortly before RT increased the loco-regional blood flow that contributed to better local oxygenation (39). A study with an orthotopic mouse model of head and neck squamous cell carcinoma (HNSCC) demonstrated that PBM does not protect the tumor from the cytotoxic effects of RT (40).

In contrast, a decreased mitotic rate was found in gingival SCC after PBM at 805 nm and energy density of 4 J/cm^2^ and 20 J/cm^2^ (25), whereas no effect on cell proliferation or protein expression of osteosarcoma cells was found when PBM was administered with a wavelength of 830 nm (41). PBM (808 nm; 5.85 and 7.8 J/cm^2^) had an inhibitory effect on the proliferation of a human hepatoma cells line (42), and a study with that glioblastoma/astrocytoma cells demonstrated a slightly decreased mitotic rate after PBM at 805 nm and 5–20 J/cm^2^ (43). Similarly, 808 nm laser irradiation with an energy density of more than 5 J/cm^2^ inhibited cell proliferation of glioblastoma cells *in vitro* (44). Moreover, a study observed growth inhibition of cancer cell lines at relatively high cumulative PBM doses (45). This prompted another study to hypothesize that PBM may have a therapeutic potential in lung cancer (46). PBM administered at a dose of 150 J/cm^2^ appeared safe, with only minor effects on B16F10 melanoma cell proliferation *in vitro*, and had no significant effect on tumor growth *in vivo*. Only a high-power density (2.5 W/cm^2^) combined with a very high dose of 1050 J/cm^2^ could induce melanoma tumor growth *in vivo* (47). Current reports from *in vitro* studies suggest that PBM may favor tumor progression for OSCC through the activation of the Akt/mTOR pathway (33), cellular proliferation (32) (34), and cellular migration (48), while there are other reports on reduction in tumor growth (48, 49). These controversial results, in addition, to being *in-vitro* evaluations, could be due to the differences in poorly documented parameters, and experimental model systems (e.g; cell confluency, media conditions, etc) (50). It is important to note that the results suggesting PBM tumor enhancement was not replicable in other studies.

PBM (660 nm, 30mW, 424 mW/cm^2^, 56.4 J/cm^2^, 133 sec, 4 J) applied to chemically-induced OSCC in hamster cheek pouch tissue increased the growth and severity of OSCC (51). However, different results were demonstrated in a mouse model, which was used to study the effects of PBM on multiple UV-induced skin tumors. The experimental mice received full-body PBM for 37 days (670 nm, twice a day, 5 J/cm^2^), whereas control animals received sham PBM. There was no enhanced tumor growth. Moreover, there was even a small but significant reduction in tumor area in the PBM group, which could be explained by a local photodynamic effect or PBM-induced antitumor immune activity (52). Similar results were reported in a rat study showing that small tumors exposed to PBM receded and completely disappeared (53). It was hypothesized that upregulation of ATP signaling by PBM promoted apoptosis and differentiation of tumor cells, thereby slowing tumor proliferation (54, 55). In an animal model of leukemia, all the animals developed chemotherapy-induced alopecia (CIA). In order to stimulate hair regrowth, rats in the experimental group received PBM, whereas control animals received sham treatment. There was no significant difference in leukemia development between the two groups, as 22% of the PBM-treated animals and 20% of the control animals remained leukemia-free. As such, PBM therapy did not negatively influence the efficacy of CT (56). A study with xenograft melanoma and OSCC mouse models demonstrated that PBM reduced tumor growth and invasiveness. This study suggests that PBM can indirectly attack tumor tissue by stimulating anti-tumor immunity and normalizing tumor vessels after PBM (49). Several clinical studies on the impact of PBM treatments on tumor burden are evidenced by their use for managing oral mucositis (57–59). These reports clearly demonstrate the reduced tumor incidences, both recurrences at the primary site and secondaries, in these patients attributed to several factors such as the ability to complete prescribed oncotherapy regimens, improved host health and resilience, and thus improved anti-tumor responses. While direct PBM contributions to these anti-tumor responses are plausible, it remains to be thoroughly investigated (60, 61).

The results from these studies suggest that different tumor cells may have distinct responses to specific PBM parameters and doses. In part, these differences may also be explained by variations in the cellular microenvironment since these have been shown to affect cellular signal transduction pathways to PBM exposure. The microenvironment of tumor cells varies among *in vitro* studies and differs significantly from that found in animal models. Moreover, it is clear that additional studies using *in vivo* systems and different tumor lines are needed to better understand the differences in tumor response to PBM and how pre-treatment molecular and genomic characterization of malignancies can be used to determine the appropriate fit for PBM like other aspects of precision medicine. Based on this current evidence, the WALT expert panel concludes that PBM is safe for use in cancer-bearing patients, but does not recommend direct treatments over the tumor site. The ‘*one-size-fits-all*’ protocols or ‘*point-and-shoot*’ approach should no longer be acceptable for PBM treatments for its broad range of clinical applications. Rationalized dosimetry, including individual wavelength photon energy (photonic fluence), appropriate delivery technique, and clinical judgment of targeted biological responses are essential for optimal clinical therapeutic outcomes (62, 63).

#### 3.2.2 Human clinical safety data

A clinical study reported no differences in cancer recurrence rates for patients receiving PBM for lymphedema following breast cancer treatment compared to controls (64). A recent RCT in which PBM was administered for prevention of OM during CRT in HNC patients (diagnosed with SCC of the nasopharynx, oropharynx, and hypopharynx) reported that at a median follow-up of 18 months (range 10-48 months), patients treated with PBM had better locoregional disease control and improved progression-free or overall survival (65). A recent retrospective analysis on 152 advanced OSCC patients examined the outcome of cancer therapy and the incidence of tumor recurrence after PBM (660 nm, 40 mW, 10 J/cm^2^) for the prevention of OM. Results showed that prophylactic PBM did not influence treatment outcome of primary cancer, recurrence, the development of new primary tumors, or survival in advanced OSCC patients (66), bearing in mind the individual tumor response in the era of precision oncology.

PBM in the red or NIR spectrum may be safe and effective in managing several complications of cancer therapy and hence should be considered for cancer patients (67). Nevertheless, as robust evidence for the lack of malignant cell protection or enhancement of tumor growth has not been published, and vigilance remains warranted. Given the lack of definitive data with respect to long-term survival and in recognition of the complexities, which govern tumor responsiveness, it is incumbent on the clinician to fully inform patients of the potential benefits and risks associated with PBM. Given its tremendous potential in the oncology population, aggressive pre-clinical and clinical investigations are critical to fully understand those parameters, which define tumor effects and patients’ response or non-responsiveness to PBM benefits.

### 3.3 Clinical indications

Virtually all conditions modulated by PBM (e.g., inflammation, ulceration, edema, pain, fibrosis, neurological and muscular injury) are thought to be involved in the pathogenesis of RT, HSCT, CT, or CRT-induced complications in patients treated for cancer.

#### 3.3.1 Acute oral mucositis

Oral mucositis (OM) is defined as an injury to the mucous lining of the oral cavity due to chemical irritations, CT, or RT. The incidence of OM is 59-100% in patients with oral or oropharyngeal cancer undergoing RT to the head and neck, and approximately 80% of the patients receiving myeloablative HSCT and high dose-conditioning regimen. OM is common in patients treated with CT for hematological cancer and occurs less frequently in patients receiving CT for solid cancers, affecting 15-80%. In recent years, mucosal injury has become prevalent in patients treated with certain types of targeted therapy and immunotherapy. Considering that the pathogenesis and course of OM depend on the clinical circumstances, the management of OM is discussed separately for each cancer type and treatment that causes it (68).

The underlying pathophysiology of OM is related to multiple factors. It consists of simultaneous, interrelated events that develop in a progressive mode in multiple tissue regions, including the epithelium and the connective tissue. As such, a five-phase model of OM was developed based on extensive research (69, 70). The key players in the development of OM are the excessive reactive oxygen species (ROS) production (71) and the activation of nuclear factor kappa B (NFκB). Studies also demonstrate that microvascular damage, the production of pro-inflammatory cytokines, host-microbiome interactions, and extracellular matrix (ECM) alterations are implicated in the pathogenesis of OM (72). Recent advances in understanding the pathogenesis of OM highlight emerging mediators of toxicity and potential insights from technological advances in mucositis research (68). This review noted that the precise etiopathology of OM induced by targeted therapy or immunotherapy requires further investigations.

Clinically OM is characterized by erythematous mucosal changes, which can develop into oral ulcerations (4, 5, 69, 73). It can significantly impair the patients’ QoL and functional status and interfere with the cancer treatment regimen. Furthermore, OM may increase the risk for bacteremia and septicemia in immunosuppressed patients and is associated with increased mortality at day 100 post-HSCT (74). Interestingly, acute OM may be associated with an increased risk of chronic mucositis in HNC patients (75, 76). Up to now, effective management strategies for OM are still scarce (77), and pain control is often insufficient (73). However, several interventions reached the level of evidence that allowed the Mucositis Study Group (MSG) of the Multinational Association for Supportive Care in Cancer/International Society of Oral Oncology (MASCC/ISOO) to recommend Clinical Practice Guidelines or suggest their use in specific patient populations (HNC, hematological cancer, solid cancers), cancer therapy modality (RT for HNC, HSCT, CT, combination of RT-CT), and for a specific purpose (prevention/treatment) (78). The reader is advised to read all the MASCC/ISOO guidelines, the methods used to develop them, and the general concepts of application.

**Figure 1:**
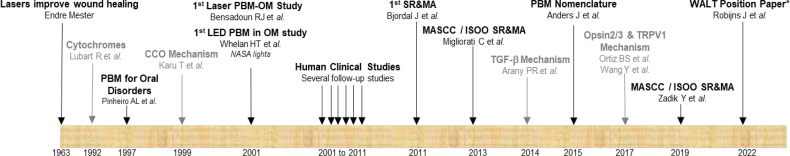
Timeline of advances in the application of PBM therapy for oral mucositis. The grey font represents basic science advances. *current WALT position paper. Abbreviations used SR&MA – Systematic review and meta-analysis; OM –Oral Mucositis; PBM - Photobiomodulation; MASCC – Multinational Association for Supportive Care in Cancer; ISOO – International Society for Oral oncology; WALT – World Association for Photobiomodulation Therapy; CCO – Cytochrome C Oxidase; TGF-β – Transforming Growth Factor beta 1; TRPV1 - Transient Receptor Potential-V1 .

PBM is one such intervention, and this section will only describe the core of the MASCC/ISOO guidelines regarding PBM (79). Since the discovery of the wound healing effects of PBM in 1963 by Dr. Endre Mester, much research has been done regarding PBM in general. In supportive cancer care, the main focus has been OM prevention and management. **Figure 1** shows a brief timeline of the advances in the field of PBM as relevant to OM. The first clinical study on OM and PBM was conducted in 2001, which was followed by many follow-up clinical trials that have enabled a significant body of literature. This led to several systematic reviews and meta-analyses with MASCC/ISOO guidelines first published in 2013 that was recently updated in 2019. The MSG of MASCC/ISOO identified strong evidence in favor of PBM to prevent OM in three categories reflecting discrete oncotherapy scenarios (**Table 1**). This strong evidence includes multiple RCTs that consistently showed significant positive results regarding OM prevention (4). For each category, recommendations were made in favor of effective protocols supported by evidence obtained in an RCT with no major study-design flaw and that was reproducible. Importantly, since there may be more than one effective protocol for each of these categories, they recommended that the specific physical PBM setting of the selected protocol be followed thoroughly for optimal results. In other words, PBM therapy outcomes will be unpredictable if the PBM settings and delivery approaches are randomly combined from various protocols. In the WALT guidelines below, we summarize our best understanding of these parameters to suggest a starting point for clinical treatments, irrespective of a specific device, and wavelength. It is well appreciated that research about new protocols, adjustments to the individual photometric features, pediatric-population specific protocols, and technical innovations of PBM devices may lead to further refinement and update of the current recommendations. There remains some uncertainty on the potential for malignant transformation when PBM is applied directly to the tumor site, which should be avoided (4). Informed consent from the patients is essential, including information about the expected benefit and potential risks of PBM should be explicitly communicated.

Further, there have been several studies directly investigating the efficacy of PBM for OM in pediatric patients with cancer of different etiologies undergoing CT, mixed RT/CRT, or mixed HSCT/CT (17, 80–96). The study settings ranged from case series, cohort studies, and non-randomized trials to RCTs. The results are promising as demonstrated in the meta-analysis by Patel et al. in 2021 (97). They showed that PBM could significantly reduce the incidence of severe OM in pediatric patients based on 16 studies. However, due to significant differences in PBM protocols in these trials (as IN the MASCC/ISOO analysis), it is hard to make definite recommendations for pediatric patients at the moment (97–100). Nonetheless, these studies emphasize the non-invasive, patient-friendly, safe and well-tolerated therapy PBM represents. Hence, we include a PBM protocol for the prevention and treatment of OM.

##### 3.3.1.1 WALT recommendation 2022: clinical practice guidelines

For Prevention of oral mucositis with an *intra-oral* device, WALT recommends a visible wavelength (630-680 nm) LED/Laser device with a power density (treatment surface irradiance) of 10-50 mW/cm^2^ for a total dose of 1.2 Einstein (photon fluence at 650 nm = 5.7 p.J/cm^2^) of per treatment field performed within 30 to 120 min prior to oncotherapy. Other wavelengths (400-1100 nm) may be used with suitable adjustment to dosing, but treatments must be monitored to ensure a non-thermal (< 45 °C) process.

For Treatments of oral mucositis with an *intra-oral* device, similar device parameters for a total dose of 2.5 Einstein (photon fluence at 650 nm = 11.4 p.J/cm^2^) should be used and repeated 3 - 4 times a week for at least 15-20 sessions or until healing after the end of oncotherapy.

For Prevention of oral mucositis with a *transcutaneous* device, WALT recommends a near-infrared wavelength (800-1100 nm) LED/Laser device with a power density (treatment surface irradiance) of 30-150 mW/cm^2^ for a total dose 1 Einstein (photon fluence at 810nm = 4.5 p.J/cm^2^) per treatment field performed within 30 to 120 min prior to oncotherapy.

For treatments of oral mucositis with a *transcutaneous* device, similar device parameters for a total dose of 6 1.3 Einstein (photon fluence at 810nm = 9 p.J/cm^2^) should be used and repeated 3 - 4 times a week for atleast 15-20 sessions or until healing after the end of oncotherapy. Other wavelengths (400-1100 nm) may be used with suitable adjustment to dosing, but treatments must be monitored to ensure they are non-thermal (< 45°C).

Consideration for Pediatric protocol: As there is inadequate data, we suggest the same protocols as in adults for both trans-cutaneous and intra-oral approaches (wavelengths, fluence per fraction, irradiance, number of treatments per week, total number of PBM treatments) be used for preventive and curative intent. We did note that the trans-cutaneous devices appear to be more clinically practical, have higher patient acceptance, and hence are easier to implement in children. Better-designed, multicenter clinical research studies using these recommendations as a starting point are essential to further optimize and validate PBM treatments for this specific application.

#### 3.3.2 Xerostomia and hyposalivation

One of the common oral complications related to therapy for HNC is hyposalivation, and its accompanying symptom is termed xerostomia (i.e., subjective sensation of dry mouth). Saliva plays a key role in oral mucosal integrity, oral caloric intake, taste perception, and speech (101). A substantial decrease in salivary function reduces QoL and increases the burden of long-term dental care (102, 103). Dry mouth affects about 74% of patients immediately after RT and increases to 85% two years after conventional RT to the head and neck area (104, 105). While IMRT may preserve some of the major salivary glands, 68% of patients develop xerostomia two years post-RT (106). Hyposalivation and xerostomia occur following CT, radioactive iodine treatment, HSCT, targeted therapy, and immunotherapy. About 40% of patients experience dry mouth during allo-HSCT, and increasing to 79% of patients at about 7 years post allo-HSCT. It is suspected that much of the late dry mouth is due to chronic graft-versus-host disease (GVHD) (105). Moreover, it may occur secondary to medications used to support the cancer patient (e.g., opioid analgesics, centrally acting pain medications) and with dehydration. Saliva is an important factor in the maintenance of mucosal integrity, promotion of oral wound healing, taste perception, formation of food bolus, initiation of food ingestion, and swallowing and speech (101). Further, it has a function in maintaining the integrity of the oral structure, in lubrication and hydration, and in modifying the bacterial metabolism and adherence to the tooth surface. Hyposalivation can negatively impact the patients’ QoL and lead to an increased risk of dental caries and tooth loss (102, 103).

A systemic review followed the methods described above, and similar to the MASCC/ISOO approach, added the reproducibility variable as an exclusion criterion. The details of this systematic review can be found in Heiskanen, 2019 (79). Briefly, six controlled clinical trials on PBM and xerostomia/hyposalivation were identified, two reported on the same patient population and were considered as a single study for the purpose of our analysis (**Table 1**). Four studies examined patients with HNC treated with RT or RT combined with CT, and one study was in patients undergoing HSCT. Considering that salivary gland damage caused by RT may be different from the damage caused by high-dose CT, each patient population is considered separately (107–112).

Regarding RT or RT-CT induced salivary gland damage, when categorizing the studies according to the aim of the PBM, there was one RCT for prevention (106, 107) and one for treatment of salivary hypofunction (109). In addition, for the prevention of salivary hypofunction in HNC patients, there was a comparative study (110), and for the treatment of salivary hypofunction in HNC patients, there was an additional before-and-after study (108). The protocols used in these studies varied regarding the laser type, the approach (extra- or intra-oral), number of sites applied, power, irradiance, time per point, fluence, and timing relative to the RT. All the studies had relatively low power, and none included placebo or were double-blind. The results for the prevention or treatment of RT/RT-CT associated salivary hypofunction were mixed with some studies demonstrating benefit in objective outcome measures (106–108) and subjective outcome measures (110), whereas other trials reported no effect for these measures (109) or for critical outcome measures (110). For patients undergoing HSCT, a single RCT assessed the effect of PBM on xerostomia (110). The primary endpoint of the study was the prevention of OM, but also data was collected for xerostomia. The study showed a significant improvement in the xerostomia score in the PBM group compared to the control group when the scores were accumulated until day 21 post-HSCT. This study did not assess objective outcome measures for salivary gland dysfunction (111).

In summary, the evidence about PBM for cancer therapy-associated salivary gland dysfunction is limited. Some evidence indicates that there is a promising potential for this therapy for the prevention or treatment of salivary gland dysfunction. More research is essential (**Table 2**).

##### 3.3.2.1 WALT recommendation 2022: expert consensus opinion

There is inadequate data to provide clinical treatment guidelines. Hence, we provide the following technical recommendations to further clinical treatments and research studies for PBM therapy in managing oncotherapy-associated xerostomia and hyposalivation. WALT recommends treatments with a transcutaneous PBM device using a visible or near-infrared wavelength (400-1100 nm) LED/Laser device with a power density (treatment surface irradiance) of 10-150 mW/cm^2^ for a total dose 2 Einstein (photon fluence at 810 nm = 9 p.J/cm^2^) per treatment field performed. Treatments should be repeated 2 to 3 times a week for at least 3 to 4 weeks, or clinical benefit is evident. It is noted that this protocol may have to be repeated after 3 to 6 months for sustained benefits. Better-designed, multicenter clinical research studies using these recommendations as a starting point are essential to further optimize and validate PBM treatments for this specific application.

#### 3.3.3 Acute dysphagia

Patients undergoing RT or CRT for HNC have an increased risk on developing dysphagia depending upon treatment volumes, which is characterized by pain and difficulty in swallowing. The prevalence of dysphagia in general oncologic patients is 15.4%. This disorder may involve changes in neuromusculoskeletal structure and function, anatomical alterations, changes in salivary flow and consistency, adverse effects during oncologic surgeries in the head and neck area, and/or odynophagia. These negatively impact the patients’ QoL and can lead to nutritional deficiencies, which can result in increased health care costs (111, 112). The pathophysiology is complex, and multiple mechanisms may overlap, which may change along the course of the disease and treatment. There is a strong relationship between OM, xerostomia, and dysphagia. Moreover, there seems to be a significant association between these oral problems and the patient’s Karnofsky level (113).

In a double-blind RCT with patients with hematologic malignancies submitted to high dose CRT followed by an autologous peripheral SCT or bone marrow transplant (BMT), patients were preventively treated with intraoral PBM for OM. A significant improvement of the ability to swallow was noted (12.8 ± 3.1 L+ vs. 19.8 ± 4.6 L-, p= 0,01), as well as improved saliva production (5.2 ± 1.3 L+ versus 16.2 ± 2.4, p= 0,005) in patients treated with PBM versus the control group (110). Two prospective RCTs with HNC patients treated with intraoral PBM have been published in which mucositis-related dysphagia was a secondary endpoint. A double-blind, phase III study did not demonstrate a reduction in the incidence of grade 3 pharyngeal dysphagia using low-dose PBM (0.1J/point) five times per week (70). However, the other RCT showed a significant reduction of dysphagia by using a combination of intraoral PBM five times per week before each RT session and total parenteral nutrition (TPN) (114). A prospective, randomized, double-blind, phase III trial with HNC patients evaluated the use of intraoral PBM for the prevention and treatment of OM. The study showed that gastrostomy need was significantly lower in PBM patients than in placebo patients (p= 0.01). The QoL of the PBM group was significantly better (64). Few PBM studies have reported dysphagia as an endpoint. As such, currently, it is difficult to provide suggestions on the use of PBM for the management of dysphagia. Therefore, it is recommended that future clinical trials need to include dysphagia as a primary endpoint.

##### 3.3.3.1 WALT recommendation 2022: expert consensus opinion

There is inadequate data to provide clinical treatment guidelines. Hence, we provide the following technical recommendations to further clinical treatments and research studies for PBM therapy in managing oncotherapy-associated acute dysphagia. WALT recommends a near-infrared wavelength 810 nm LED/Laser device with a power density (treatment surface irradiance) of 30-150 mW/cm^2^ for a total dose 1 Einstein (photon fluence at 810 n = 4.5 p.J/cm^2^) per treatment field. Treatments may be repeated 2 -3 times a week, for at least 3 to 4 weeks or till improvements are evident. Better-designed, multicenter clinical research studies using these recommendations as a starting point are essential to further optimize and validate PBM treatments for this specific application.

#### 3.3.4 Acute radiation dermatitis

Up to 95% of the patients undergoing RT develop acute radiodermatitis (ARD), which is an inflammatory skin reaction inflicted by cellular injury due to ionizing radiation. Skin reactions typically become visible two to three weeks after the first RT session and can be graded based on the criteria of the Radiation Therapy Oncology Group (RTOG), from erythema, and dry desquamation (grade 1), patchy moist desquamation (grade 2), confluent moist desquamation (grade 3), or in rare cases necrosis with hemorrhage and ulceration can occur (grade 4) (115, 116). The severity of ARD is dependent on several patient and treatment-related characteristics (117, 118).

The pathophysiology of ARD is complex, and the severity depends on the survival of the actively proliferating basal cells in the epidermis. In the first phase, an erythematous skin reaction develops caused by vascular damage leading to an increase in vascular permeability and vasodilation. This is followed by an inflammatory reaction characterized by transendothelial migration of circulatory immune cells to the irradiated skin driven by the production of cytokines and chemokines. The skin compensates by up-regulating the proliferation of the basal epidermal stem cells, leading to dry desquamation when the turnover of the new cells is faster than the shedding of the old ones. When the basal stem cells become depleted, moist desquamation arises (119). ARD is a distressing and painful side effect of RT, which can lead to problems in the patients’ daily life, negatively affecting their QoL. In cases of severe ARD, interruption of RT can be necessary, which can negatively affect the treatment outcome and overall patient survival (120). Guidelines for prevention and management of ARD put forth by MASCC recommend the implementation of daily hygiene practices and the application of potent topical steroids (120–123).

The use of PBM for the prevention and management of ARD was first reported in a case-control study. Three breast cancer patients with RT-induced skin ulcers were treated with PBM, which demonstrated improved wound healing (124, 125). A prospective intervention trial with a retrospective control group showed that PBM reduced the incidence of grade 2≥ ARD in breast cancer patients (126). In contrast, an RCT was not able to replicate these results (127). In a prospective, quasi-experimental intervention trial (DERMIS) with breast cancer patients undergoing RT, PBM was applied twice per week in a therapeutic manner. Results demonstrated that PBM prevented the aggravation of ARD and improved the patients’ QoL towards the end of RT (128). In a prospective intervention trial with breast cancer patients, they applied PBM from the start of RT twice per week and showed that PBM was associated with a reduced incidence of severe ARD (129). The TRANSDERMIS trial was a patient-blinded RCT investigating the use of PBM in the prevention of ARD in breast cancer patients. PBM was applied twice weekly during the full course of RT. The trial showed that PBM was able to prevent the development of grade 2≥ ARD both by subjective and objective outcome measures and, the QoL measures were significantly better in the PBM treated group (130, 131). A recent RCT demonstrated that PBM improved ARD in HNC patients (132). The DERMISHEAD study (ClinicalTrials.gov Identifier: NCT02738268) is an actively enrolling placebo-controlled RCT, investigating the efficacy of PBM in preventing ARD in HNC patients (133).

##### 3.3.4.1 WALT recommendation 2022: expert consensus opinion

There is inadequate data to provide clinical treatment guidelines. Hence, we provide the following technical recommendations to further clinical treatments and research studies for PBM therapy in managing oncotherapy-associated radiation dermatitis. WALT recommends treatments with a transcutaneous PBM device using a visible or near-infrared wavelength (400-1100 nm) LED/Laser device with a power density (treatment surface irradiance) of 10-150 mW/cm^2^ for a total dose of 1 Einstein (photon fluence at 810 nm = 4.5 p.J/cm^2^) treatment field performed within 30 to 120 min prior to oncotherapy. Treatments should be repeated 3 - 4 times a week for at least 5 to 6 weeks, or clinical benefit is evident. When ARD is associated with inflammation of subcutaneous tissue, WALT recommends using a near-infrared (730 – 800 nm) transcutaneous LED/laser device with a dose 2 Einstein (photon fluence at 810 nm = 9 p.J/cm^2^) per treatment field 3-4 times a week, for at least 5 to 6 weeks. Better-designed, multicenter clinical research studies using these recommendations as a starting point are essential to further optimize and validate PBM treatments for this specific application.

#### 3.3.5 Lymphedema

As a consequence of cancer treatment, Lymphedema is apparent in breast cancer and HNC patients HNC) (134, 135). About 20% of breast cancer patients can develop lymphedema in the upper extremity after cancer treatment (134). In the case of HNC, it is often an undervalued late effect, but it has been diminished with the introduction of Intensity-Modulated Radiation Therapy (IMRT). In HNC patients, lymphedema may develop externally, on the face and neck, and/or internally involving the larynx and pharynx. External lymphedema may negatively impact the patients’ body image, while internal lymphedema may disturb the normal breathing process, lead to dysphagia and trismus, and impact speech. The incidence of lymphedema in HNC patients is relatively high. For example, a single-center study on 81 HNC patients reported an incidence of 75%, with 10% external, 39% internal, and 51% experiencing both types of lymphedema. Pharyngeal carcinoma patients had the highest risk. Chronic lymphedema that develops later (2–6 months after) may resolve spontaneously in some patients, but not in all (135). The pathobiology of lymphedema consists of an initiation where disruption of lymphatic structures occurs by surgery or RT, leading to the accumulation of lymph fluid in the interstitial tissues. Inflammatory cells will infiltrate the affected tissue. This inflammatory reaction will be enhanced by the recruitment of additional immune cells from the circulation by cytokines and chemokines that remain in the tissue due to lymphatic dysfunction. This will ultimately lead to additional soft tissue damage and fibrosis, worsening the lymphatic function even more (134, 136).

The current treatment of lymphedema is based on symptom management and prevention of disease progression. As such, the main therapeutic option for lymphedema is complete decongestive therapy (CDT) (137–139). There was only one case-control study that showed a beneficial effect of PBM in the management of lymphedema in HNC patients (140). However, no prospective trial has been published to date, and further research is recommended. Also, in breast cancer patients, PBM has been investigated as a potential treatment for post-mastectomy lymphedema. The possible underlying mechanism of PBM for this indication is the stimulation of lymphangiogenesis, enhancement of lymphatic motility, and reduction of lymphatic fibrosis. A meta-analysis showed moderate evidence for the effectiveness of PBM in the reduction of arm swelling and pain in women with breast cancer-related lymphedema (98). Additionally, results demonstrated that the combination of PBM with CDT was more effective in reducing the arm volume than with CDT alone (141–144). An RCT with 40 breast cancer patients demonstrated a pain decrease of 50% and significantly higher grip strength after PBM (145). Another RCT evaluated the effectiveness of PBM as a complementary treatment to CDT for managing lymphedema in breast cancer patients 12 months post-intervention. Results demonstrated that the combination of PBM and CDT significantly decreased the number of lymphedema symptoms and relieved their impaired limb mobility symptoms. In addition, PBM reduced their emotional distress from lymphedema symptoms, such as sadness and self-perception (146).

##### 3.3.5.1 WALT recommendation 2022: expert consensus opinion

There is inadequate data to provide clinical treatment guidelines. Hence, we provide the following technical recommendations to further clinical treatments and research studies for PBM therapy in managing oncotherapy-associated lymphedema. WALT recommends treatments with a transcutaneous PBM device using a visible or near-infrared wavelength (400-1100 nm) LED/Laser device with a power density (treatment surface irradiance) of 10-150 mW/cm^2^ for a total dose of 2 Einstein (photon fluence at 810 nm = 9 p.J/cm^2^) per treatment field. Treatments should be repeated 2 to 3 times a week for at least 3 to 4 weeks, or clinical benefit is evident. Better-designed, multicenter clinical research studies using these recommendations as a starting point are essential to further optimize and validate PBM treatments for this specific application.

#### 3.3.6 Dysgeusia

Dysgeusia is a taste disorder characterized by a persistent gustatory sensation in the absence of taste stimulants or distorted gustatory perception. The complete loss of gustatory perception (ageusia) is rare, as most of the patients experience a reduction in the intensity of gustatory perception (hypogeusia). This disorder may be temporary or, in some cases, permanent (147, 148). Taste dysfunction in cancer patients can lead to decreased food intake and subsequent malnutrition and weight loss, resulting in a decreased QoL and increased healthcare costs (149, 150). In one report, the prevalence of dysgeusia in patients submitted to induction CT exclusively was 56.3%, to RT exclusively 66.5%, and to CRT 76%. About 15% of the patients continued to experience this side effect up to one-year post-treatment (151).

The pathogenesis of dysgeusia is complex and not well understood because there are multiple mechanical and chemical factors and clinical conditions (systemic factors and diseases; hyposalivation; oral, dental, and oropharyngeal pathologies) that can impact the gustatory perception (152). The mechanism by which CT and RT cause taste alterations is believed to be due to neurological damage, a decrease in the number of receptor cells, and/or an alteration of cell structure (151). Evaluating the severity of dysgeusia is complicated because of the heterogeneity of the methods, which can be qualitative, sip and spit tests, and/or patient surveys (150). No standard management approaches for dysgeusia have been established (151).

Other than a single case report with a positive outcome (153), there are no prospective clinical trials evaluating the use of PBM in the management of dysgeusia. Therefore, the potential utility of PBM in the management of dysgeusia in cancer patients currently remains limited.

##### 3.3.6.1 WALT Recommendation 2022: Expert Consensus Opinion

There is inadequate data to provide clinical treatment guidelines. Hence, we provide the following technical recommendations to further clinical treatments and research studies for PBM therapy in managing oncotherapy-associated dysgeusia. WALT recommends treatments with an intraoral visible (red 630-680 nm) or transcutaneous near-infrared (800-1100 nm) wavelength LED/laser device with a power density (treatment surface irradiance) of 10-150 mW/cm^2^ for a total dose of 2 Einstein (photon fluence at 810 nm = 9 p.J/cm^2^) per treatment field performed. Treatments should be repeated 3 to 4 times a week for 3 to 4 weeks, or clinical benefit is evident.Better-designed, multicenter clinical research studies using these recommendations as a starting point are essential to further optimize and validate PBM treatments for this specific application.

#### 3.3.7 Trismus

Trismus is a restriction in jaw movement, which may be due to tumor, local infection, tissue fibrosis, pain upon mouth opening or a tonic contraction of the muscles of mastication (131). It has been defined variously as a mouth opening of less than 40 or less than 20 mm, whereas less restrictive classifications also have been used (154). Trismus is caused by tumor invasion or RT of the masticatory muscles or the temporomandibular joint (154, 155). The weighted prevalence of trismus is estimated to be 25% following conventional RT, 5% following IMRT, and 31% for CRT (156). The risk of trismus increases when the cumulative radiation dose is higher than 60 Gy (157). However, the most important risk factor is the inclusion of the lateral pterygoid muscles in the high-dose RT field (158). Trismus typically develops 3-6 months post-RT and frequently becomes a lifelong problem (155, 159).

As demonstrated by several studies, fibrosis seems to be an important event in the development of RT-induced trismus. Other factors enhancing the development of trismus are post-surgical scar tissue, nerve damage, or a combination of these factors (155). Mandibular hypomobility eventually leads to muscle shortening and possibly temporomandibular joint dysfunction (156). The number of negative consequences of trismus and orofacial pain for the patients’ general health is high, including a reduction in nutritional intake, difficulty speaking, compromised oral health, and a poor QoL (160). Trismus can be prevented by avoiding RT to the masticatory structures. However, early interventions can also be used to minimize trismus (161–163).

There is a single case report study that investigated the use of PBM in the management of trismus two months post-RT. At the final PBM session, the patient’s mouth opening increased from 20 to 30 mm, and the pain decreased from a visual analog score (VAS) from 9 to 1. These positive results persisted up to one year after the final PBM session (164). The main rationale for a possible clinical benefit of PBM in the management of trismus is the potential of PBM to reduce fibrosis and promote muscle regeneration. Due to the limited amount of clinical data, no recommendations are possible on the use of PBM for the management of trismus.

##### 3.3.7.1 WALT Recommendation 2022: Expert Consensus Opinion

There is inadequate data to provide clinical treatment guidelines. Hence, we provide the following technical recommendations to further clinical treatments and research studies for PBM therapy in managing oncotherapy-associated trismus. WALT recommends treatments with a transcutaneous near-infrared (800-1100 nm) wavelength LED/laser device with a power density (treatment surface irradiance) of 10-150 mW/cm^2^ for a total dose of 2 Einstein (photon fluence at 810 nm = 9 p.J/cm^2^) per treatment field performed. Treatments should be repeated 3 to 4 times a week for 4 to 6 weeks, or clinical benefit is evident.Better-designed, multicenter clinical research studies using these recommendations as a starting point are essential to further optimize and validate PBM treatments for this specific application.

#### 3.3.8 Bone Necrosis

Bone necrosis can occur due to RT of the HNC region, which is specified as osteoradionecrosis (ORN), or due to specific medication, also termed medication-related osteonecrosis of the jaw (MRONJ) (165, 166) (167, 168). RT can cause vascular occlusion leading to the loss of osteocytes, which can result in bone necrosis (165, 166). Mandibular ORN prevalence is estimated between 5%-15%, but due to the improvements in the RT technique and the introduction of IMRT, less than 5% of patients develop ORN. Moreover, suitable pre-RT dental care can also help to prevent ORN (155, 169). To date, the underlying mechanism of ORN is still not completely clear. Most obvious, RT induces a fibro-atrophic process, which will consist of free radical formation, endothelial dysfunction, inflammation, microvascular thrombosis, fibrosis, and remodeling. This will eventually result in bone and tissue necrosis (170). Several factors can increase the risk of ORN, including inflammatory dental disease, soft tissue trauma, and dental surgical procedures to the bone in sites of high dose RT. An important risk factor of ORN is removing diseased teeth after RT. However, bone necrosis can also develop due to periodontal disease, trauma, or in a spontaneous manner (171, 172). Removing compromised teeth before RT and proper dental care during and following RT is essential to prevent ORN (165, 166).

Medication-related osteonecrosis of the jaw (MRONJ) can occur in cancer patients undergoing treatment with angiogenesis inhibitors (e.g., bevacizumab, sunitinib) for advanced HNC and in patients with bone metastases treated with bisphosphonates (167, 168). Bevacizumab is an antibody that blocks vascular endothelial growth factor (173), which can lead to tissue ischemia (174–176). In addition, it may inhibit proper wound healing and stimulate oral mucosal breakdown leading to exposure of the necrotic jawbone (177). Sunitinib is a TKI that can also cause MRONJ by blocking several pathways related to angiogenesis (178, 179). Bisphosphonates inhibit bone turnover by inducing osteoclastic apoptosis and inhibiting osteoblast-mediated osteoclastic activity (168). The incidence of MRONJ ranges from 0.8% to 12%. The American Association of Oral and Maxillofacial Surgeons (AAOMS) recommends good oral hygiene, the use of pharmacological therapy (e.g., antibiotics, pain medication), and, in case of persisting exposed bone, surgical removal (180).

In an *in vivo* study, PBM applied on rat bone before and during RT had a positive effect (181), and comparable results were described by another animal study (182). However, another *in vivo* study found that PBM was not able to repair the RT-induced bone damage (183). To our knowledge, no clinical studies on the effects of PBM for ORN have been conducted. Although, several studies demonstrated a beneficial effect of PBM in the management of MRONJ induced by bisphosphonates (184–190). A review concluded that the use of PBM might have a significant advantage above classical therapy as the overall complete response rate was 55% for the patients that underwent PBM, while this was only 30% for the patients receiving the classical care (191). A study in a rodent wound healing model found evidence that both laser and LED were capable of stimulating angiogenesis *in vivo* (192). Up to now, no clinical trials on the management of MRONJ induced by angiogenesis inhibitors have been found in the literature. Rapid repair of chronic soft tissue necrosis in previously radiation-treated HNC patients has been reported in 2-4 weeks of PBM in a small series of cases (193). This finding was confirmed by similar findings in another case report of another institution (194). This case report describes an additional case of a persisting radiation-associated mucosal necrotic lesion, treated at another institution with rapid and early improvement and complete resolution in 6 weeks, comparable to the three cases previously reported (195). These cases provide clinical evidence of rapid repair of radiation-induced chronic mucosal ulceration due to PBM.

##### 3.3.8.1 WALT Recommendation 2022: Expert Consensus Opinion

There is inadequate data to provide clinical treatment guidelines. Hence, we provide the following technical recommendations to further clinical treatments and research studies for PBM therapy in managing oncotherapy-associated bone necrosis. WALT recommends treatments with an intraoral visible (red 630-680 nm) or transcutaneous near-infrared (800-1100 nm) wavelength LED/laser device with a power density (treatment surface irradiance) of 10-150 mW/cm^2^ for a total dose of 2 Einstein (photon fluence at 810 nm = 9 p.J/cm^2^) per treatment field performed. Treatments should be repeated 3 to 4 times a week for 4 to 6 weeks, or clinical benefit is evident. Better-designed, multicenter clinical research studies using these recommendations as a starting point are essential to further optimize and validate PBM treatments for this specific application.

#### 3.3.9 Voice and Speech Alterations

The main communication tools of a person are his/her voice and speech, and they play an important role in a person’s identity and personality. The quality of the voice is determined by the movement and characteristics of the vocal cords, while the quality of speech is dependent on the resonance characteristics of the vocal tract. Any alteration to the muscle or tissue properties of the articulator structures can affect the coordinated volitional movements leading to speaking problems. Impairments of the voice and speech can significantly diminish the patient’s QoL. However, these complications do not receive much supportive care during cancer therapy. In addition, they are likely under-reported in efforts to preserve organ function after cancer therapy (196, 197).

The pathophysiology of voice and speech problems resembles that of dysphagia, which may include neuromuscular weakness due to tumor invasion. Mucositis of the soft palate and laryngeal soft tissues, fibrosis or vocal fold atrophy, edema and atrophy of laryngeal and pharyngeal tissues, and altered saliva or xerostomia can lead to CRT-induced voice and/or speech alterations (198, 199). Long-term functional impairment of the voice and/or speech may be prevented by new RT delivery techniques, including IMRT, which are designed to spare anatomical structures that are involved with voice and/or speech. Further, early speech rehabilitation may also help (200).

Currently, there are no exact figures of the prevalence of speech and voice dysfunction in advanced HNC patients treated with (C)RT. Therefore, prospective studies are needed that will include baseline measurements and standardized multidimensional assessment of functional aspects of voice and speech (196). To our knowledge, there are no studies on the effect of PBM on the quality of speech and voicing in HNC patients. Still, PBM may preserve the function of the anatomical structures involved directly and could have indirect benefits by decreasing hyposalivation.

##### 3.3.9.1 WALT Recommendation 2022: Expert Consensus Opinion

There is inadequate data to provide clinical treatment guidelines. Hence, we provide the following technical recommendations to further clinical treatments and research studies for PBM therapy in managing oncotherapy-associated voice and speech alterations. WALT recommends treatments with a transcutaneous near-infrared (800-1100 nm) wavelength LED/laser device with a power density (treatment surface irradiance) of 10-150 mW/cm^2^ for a total dose of 2 Einstein (photon fluence at 810 nm = 9 p.J/cm^2^) treatment field performed. Treatments should be repeated 3 to 4 times a week for 2 to 4 weeks, or clinical benefit is evident. Better-designed, multicenter clinical research studies using these recommendations as a starting point are essential to further optimize and validate PBM treatments for this specific application.

#### 3.3.10 Palmar-plantar erythrodysesthesia

Palmar-Plantar Eythrodysesthesia (PPE), also known as hand-foot syndrome, is a side effect of many classic CT agents and newer molecular targeted therapies (201, 202). PPE is characterized by redness, swelling, and pain on the palms of the hands and/or the soles of the feet, that when severe, can progress to frank blistering. It sometimes occurs elsewhere on the skin, such as the knees or elbows, but this is less common (201, 202). The most conventional cytotoxic drugs that can cause this syndrome include doxorubicin, cytarabine, docetaxel, capecitabine, or 5-fluorouracil. Also, multitargeted TKIs such as sorafenib, sunitinib, regorafenib, and others that target angiogenesis is associated with PPE. PPE is usually the worst during the first 6 weeks of treatment with targeted therapy, although with conventional CT, the condition may not present until 2-3 months after initiation of therapy (203–205).

The underlying mechanism is still not clear. There are several factors that could explain the development of PPE in hand palms and foot soles, such as the rapid cell division, gravitational forces, specific vascular anatomy, temperature gradients, as well as increased drug concentration in the eccrine sweat glands (203–205). Management of PPE consists of discontinuation of the drug and symptomatic treatment to provide relief, diminish edema, and prevent superinfection. Symptom management includes wound care, pain medication, and/or the application of topical alcohol-free (anti-inflammatory) emollients (206).

The evidence regarding the use of PBM for the management of PPE is limited. A single clinical trial with patients that presented PPE due to active CT or targeted therapy was conducted. Patients served as their own control, as one body site was treated with PBM while the other one was treated with sham laser. Preliminary results of 31 patients demonstrated no significant effect of PBM on the severity of PPE when both body sites were compared at the end of the therapy. However, patients’ pain decreased, and patient satisfaction was higher due to PBM (207).

##### 3.3.10.1 WALT Recommendation 2022: Expert Consensus Opinion

There is inadequate data to provide clinical treatment guidelines. Hence, we provide the following technical recommendations to further clinical treatments and research studies for PBM therapy in managing oncotherapy-associated Palmar-Plantar Eythrodysesthesia. WALT recommends treatments with an intraoral visible (red 630-680 nm) or transcutaneous near-infrared (800-1100 nm) wavelength LED/Laser device with a power density (treatment surface irradiance) of 10-150 mW/cm^2^ for a total dose of 2 Einstein (photon fluence at 810 nm = 9 p.J/cm^2^) per treatment field performed. For early lesions, a LED/Laser device with a red wavelength (630-660 nm) in direct contact with the skin can also be used. Treatments should be repeated 2 to 3 times a week for 2 to 4 weeks, or clinical benefit is evident. Better-designed, multicenter clinical research studies using these recommendations as a starting point are essential to further optimize and validate PBM treatments for this specific application.

#### 3.3.11 Graft versus host disease

One of the complications that occur following allogenic HSCT is graft-versus-host disease (GVHD). This side effect is accompanied by skin, digestive, and oral problems. The mechanism underlying GVHD is based on an immune reaction caused by the immune cells from the non-identical donor (the graft) that recognize the transplant recipient (the host) as foreign. When it occurs in the first 100 days after transplantation, it is defined as the acute form, while after this time period, it is called chronic GVHD, which can persist for months to years. Oral problems occur mostly during the chronic GVHD and are characterized by white and red changes on oral mucosa with or without ulceration and may be complicated by dry mouth. Moreover, oral lichen planus GVHD can occur as a single manifestation of GVHD, which often resembles classic oral lichen planus, an inflammatory condition that affects oral mucous membranes. Oral lichen planus is characterized by white, lacy patches; red, swollen tissues; or open ulcers. Clinical symptoms range from a painful, burning sensation, a diminished taste, a loss of appetite, and hyposalivation in some extreme cases (208, 209).

Acute GVHD may appear in 20%–40% of patients receiving an allogeneic HSCT from a sibling with an identical human leukocyte antigen (HLA) and in over 50% of patients receiving an allogeneic HSCT from an unrelated donor. The incidence of chronic GVHD ranges from 30% to 70% (210–212). Management of oral GVHD is based on good oral hygiene and/or systemic/local treatment with steroids or other immunomodulatory medicines. However, this seems to be insufficient in some cases (208, 209). PBM has been evaluated in the treatment of oral lichen planus with positive outcomes (213–220). Based upon this, PBM has been utilized for local treatment of oral GVHD in patients with continuing symptoms and signs despite systemic and topical therapies with corticosteroids and other immunosuppressants. Two reports of patients who were treated with PBM showed mucosal lichenoid change; particularly, ulceration and erythema were improved in 3-4 weeks of treatment and oral pain, and dry mouth improved in most patients (221, 222). These findings suggest that PBM may represent an additional approach for the management of oral GVHD and suggest that controlled studies should be conducted to confirm the efficacy of PBM therapy in oral GVHD and to determine optimal PBM therapy protocols.

##### 3.3.11.1 WALT Recommendation 2022: Expert Consensus Opinion

There is inadequate data to provide clinical treatment guidelines. Hence, we provide the following technical recommendations to further clinical treatments and research studies for PBM therapy in managing oncotherapy-associated Graft-versus-Host Disease. WALT recommends treatments with a transcutaneous near-infrared (800-1100 nm) wavelength LED/laser device with a power density (treatment surface irradiance) of 10-150 mW/cm^2^ for a total dose of 2 Einstein (photon fluence at 810 nm = 9 p.J/cm^2^) per treatment field performed. For early lesions, a LED/Laser device with a red wavelength (630-660 nm) in direct contact with the skin has been noted to be beneficial as well. Treatments should be repeated 3 to 4 times a week for 4 to 6 weeks, or clinical benefit is evident.Better-designedd, multicenter clinical research studies using these recommendations as a starting point are essential to further optimize and validate PBM treatments for this specific application.

#### 3.3.12 Peripheral neuropathy

Chemotherapy-induced peripheral neuropathy (CIPN) is a common side effect of CT, with an incidence of 68% in the first month after CT (223). The main neurotoxic CT agents are taxanes, platinum drugs, vinca alkaloids, thalidomide, and bortezomib. Symptoms related to CIPN are typically symmetric and bilateral and include paresthesia, numbness, burning pain, loss of temperature sensation, and loss of tendon reflexes typically appearing in distal extremities, indicating increased vulnerability of neurons with the longest axons (224, 225). Sensory neurons are particularly affected, while motor, autonomic, or CNS involvement is rare. This selective vulnerability likely relates to the permeability of the blood-nerve barrier at the level of the dorsal root ganglion (226). CIPN impairs patients’ daily activities because of comorbidities such as psychological distress, fall risk, and poor sleep quality resulting in a significant decrease in QoL (227). Furthermore, CIPN represents a heavy economic burden (228). The pathogenesis of CIPN can be explained by the fact that neurotoxic CT agents cause mitochondrial DNA damage, stabilize or destabilize microtubule formation, or have an anti-angiogenic effect. However, the exact pathophysiology of CIPN is still not clear (226).

Pharmacological symptom management (e.g., anti-depressants or pain medication) provides a limited benefit (229, 230). In severe cases, CT dose delays and/or reductions are necessary, which can affect treatment outcomes (224). There are no widely accepted evidence-based measures to prevent or minimize CIPN (231). Several animal studies have been performed to investigate the effectiveness of PBM in the management of CIPN (232–234). They demonstrated that PBM is able to reduce neuropathic pain, promote functional recovery of peripheral nerves, and stimulate axonal growth and regeneration. Moreover, an *in vivo* study showed a reduction of cold and mechanical allodynia after PBM (233). Furthermore, research with beneficial results has been reported for PBM in patients with diabetic neuropathy, demonstrating that the nerve conduction velocity significantly improved after PBM (230, 235–237).

Three clinical trials have been conducted investigating the effect of PBM on the management of CIPN. A single group prospective trial with breast cancer patients observed a decreased Brief Pain Index (BPI) after PBM (238). According to an RCT, PBM induced a significant reduction in the modified Total Neuropathy Score (mTNS) in patients with cancer from different etiologies (239). Another prospective, single-arm study with gastrointestinal cancer patients observed a significant reduction in neurotoxicity symptoms after PBM (240, 241).

##### 3.3.12.1 WALT Recommendation 2022: Expert Consensus Opinion

There is inadequate data to provide clinical treatment guidelines. Hence, we provide the following technical recommendations to further clinical treatments and research studies for PBM therapy in managing oncotherapy-associated peripheral neuropathy. WALT recommends treatments with a transcutaneous near-infrared (800-1100 nm) wavelength LED/laser device with a power density (treatment surface irradiance) of 10-150 mW/cm^2^ for a total dose of 2 Einstein (photon fluence at 810 nm = 9 p.J/cm^2^) per treatment field performed. Treatments should be repeated 3 to 4 times a week for 4 to 6 weeks, or clinical benefit is evident. Better-designed, multicenter clinical research studies using these recommendations as a starting point are essential to further optimize and validate PBM treatments for this specific application.

#### 3.3.13 Radiation-induced fibrosis

The soft tissue and lymphatic complications of HNC radiation are ubiquitous, as more than 50% of the patients develop severe fibrosis (242). Radiation-induced fibrosis (RIF) can adversely impact patients’ QoL. A particularly important manifestation of RIF includes impaired swallowing and aspiration due to mucosal and stromal fibrosis of the neck and pharyngeal musculature, laryngeal lymphedema, and stiffness of the tissue architecture that result in substantial physical symptom burden and functional loss (135). The extent of RIF depends on multiple factors, including the tumor site, the affected adjacent organs, and the dose prescribed (243).

The underlying mechanism of RIF in normal tissues has been extensively studied. Ionizing radiation generates ROS and nitrogen species (NO) that lead to localized inflammation, which in the acute setting causes irritation of the skin and the epithelial lining of adjacent organs (70). The acute inflammatory process evolves into a chronic inflammatory state and tissue remodeling several months after RT, which can progress over many years, causing lymphedema, fibrosis, pain, atrophy, and organ dysfunction (243, 244). Transforming growth factor-beta (TGF-β) serves as one of the primary mediators in this response along with a host of other cytokines and growth factors (243). The current treatment of RIF involves taking pentoxifylline/vitamin E primarily daily for several years with modest benefit. Despite elucidation of molecular mechanisms of RIF secondary to RT in animal models, to date, no effective treatment measures are available (245).

The use of PBM in the management of fibrosis has been investigated in studies *in vitro* (246–249). The available evidence demonstrates that PBM can regulate the main targets of the fibrosis pathway ranging from the reduction of the fibroblast proliferation and migration speed, inhibition of the production of TGF-β and the related pathway, to the downregulation of the production and deposition of collagen (247). There are currently no published clinical studies using PBM to address RIF (250). Currently, investigators at New York University are evaluating the feasibility, safety, and tolerability of providing PBM for the treatment of RIF in HNC patients treated with RT. Moreover, they are investigating the corresponding metabolic changes in the microenvironment.

##### 3.3.13.1 WALT Recommendation 2022: Expert Consensus Opinion

There is inadequate data to provide clinical treatment guidelines. Hence, we provide the following technical recommendations to further clinical treatments and research studies for PBM therapy in managing radiation-induced fibrosis.

for prevention with a transcutaneous device, WALT recommends a near-infrared wavelength (800-1100 nm) LED/Laser device with a power density (treatment surface irradiance) of 10-150 mW/cm^2^ for a total dose of 2 Einstein (photon fluence at 810 nm = 9 p.J/cm^2^) per treatment field. Treatments should be performed 30 to 120 min before each treatment for the duration of the radiation treatment,

for treatments with a transcutaneous device, WALT recommends a near-infrared wavelength (800-1100 nm) LED/Laser device with a power density (treatment surface irradiance) of 10-150 mW/cm^2^ for a total dose of 2 Einstein (photon fluence at 810 nm = 9 p.J/cm^2^) per treatment field. Treatments should be repeated 3 to 4 times a week for 6 to 8 weeks, or clinical benefit is evident.

Better-designed, multicenter clinical research studies using these recommendations as a starting point are essential to further optimize and validate PBM treatments for this specific application.

#### 3.3.14 Chemotherapy-induced alopecia

Chemotherapy-induced alopecia (CIA) is a common side effect of CT, affecting approximately 65% of patients undergoing CT, although this figure is highly dependent on the CT agent used (251). CIA is a direct effect of the cytotoxic CT on the rapidly dividing cells, including the hair matrix cells (251). This side effect is considered as one of the most distressing and traumatizing experiences for cancer patients, particularly in women (252, 253). During and even after CT, CIA will hinder patients’ QoL and their ability to cope effectively with their disease. Since hair is an important indicator of femininity, attractiveness, and personality, loss of hair could lead to body dissatisfaction and poor post-treatment adjustment (254). In addition, CIA can induce physical pain such as headaches and pain on the scalp (255). There are no approved pharmacologic therapies for CIA (251). The effect of topical or oral application of minoxidil has been investigated with limited success (256, 257). The only available preventive measure is based on scalp cooling (258). Nevertheless, this treatment has a highly variable success rate and brings along certain complications (e.g., feeling cold, dry skin, nausea, mild headaches) (258).

Concerning the use of PBM and hair loss, positive results were demonstrated in clinical trials applying PBM for the treatment of androgenetic alopecia and alopecia areata (259–261). An *in vivo* study investigated the effectiveness of PBM in CIA in rats. By 15 days post-CT, the PBM-treated rats showed hair regrowth while none of the sham-treated rats did. PBM probably stimulated the proliferation of hair-matrix keratinocyte stem cells or activated dermal papilla cells (56). No clinical trials on the use of PBM for CIA are available.

##### 3.3.14.1 WALT Recommendation 2022: Expert Consensus Opinion

There is inadequate data to provide clinical treatment guidelines. Hence, we provide the following technical recommendations to further clinical treatments and research studies for PBM therapy in managing chemotherapy-induced alopecia. WALT recommends treatments with a transcutaneous visible to near-infrared (400-1100 nm) wavelength LED/laser device with a power density (treatment surface irradiance) of 10-150 mW/cm^2^ for a total dose of 2 Einstein (photon fluence at 810 nm = 9 p.J/cm^2^) per treatment field performed. The red (630-680 nm) wavelength has shown the most efficacy in several studies. Treatments should be repeated 3 to 4 times a week for 6 to 8 weeks, or clinical benefit is evident. Better-designed, multicenter clinical research studies using these recommendations as a starting point are essential to further optimize and validate PBM treatments for this specific application.

## 4 Conclusions and future perspectives

Cancer therapy is still associated with a wide range of acute and late complications that impair the patients’ QoL. Based on the evidence collected in this review, PBM has the potential to become a new preventive and/or therapeutic option for a broad range of acute and chronic side effects associated with cancer therapy. The effectiveness of PBM for the prevention and management of OM has already been demonstrated. As such, PBM has been taken up in the general treatment guidelines developed by the MASCC/ISOO, the European Society for Medical Oncology (ESMO), and the National Institute for Health and Care Excellence (NICE) (4, 262, 263).

The aim of this position paper is to provide scientific evidence for the use of PBM in various cancer therapy-related side effects, and a rigorous effort is made to indicate specific PBM parameters (**Table 3**). Future investigations should be performed to better define optimal PBM parameters (irradiation and treatment) for each of the complications. Concerning the safety of PBM in oncologic patients, *in vitro*, *in vivo*, and even recent clinical data have been generated. Even though the clinical trial results did not demonstrate adverse events associated with PBM, it is still necessary to treat cancer patients with caution when applying PBM. More *in vivo* studies with animal tumor models need to be developed to elucidate the effect of PBM on the tumor and its microenvironment. Large clinical trials with a wide variety of cancer patients in which different PBM parameters are tested, and that include a long follow-up period of at least five years, are required to conclude that PBM does not negatively affect cancer progression and overall survival.

An emerging approach in general medicine, which also seems to be important when applying PBM in cancer patients, is precision medicine. It is based on taking into account the patient’s personal lifestyle, environment, and variability in gene expression. Each cell type and especially tumor cells may have different responses to certain PBM parameters and doses, which are caused by variations in the cellular microenvironment. As such, more personalized PBM protocols for each indication will be necessary for the future. PBM in the daily clinical oncology practice setting may address a number of significant and common adverse effects (264). This may eventually reduce the incidence, duration, and severity of these devastating effects. Thereby, the patient will experience less pain and discomfort during and after their cancer therapy, which enables them to perform their daily activities during active cancer treatment and throughout survival. This will eventually result in improved patients’ QoL and optimization of their specific cancer treatment. Further, the treatment compliance of the patient will increase, resulting in an improved success rate of the cancer therapy. Thus, patient care will advance, which will ultimately result in increased patient survival.

## Data availability statement

The raw data supporting the conclusions of this article will be made available by the authors, without undue reservation.

## Funding

MRH was supported by US NIH Grants R01AI050875 and R21AI121700.

## Acknowledgments

The authors want to thank Laure Alston, James Carroll, Vedang Kothari, and Damien Vila for their contributions during WALT 2018 Congress.

## Conflict of interest

MRH declares the following potential conflicts of interest. Scientific Advisory Boards: Transdermal Cap Inc, Cleveland, OH; BeWell Global Inc, Wan Chai, Hong Kong; Hologenix Inc. Santa Monica, CA; LumiThera Inc, Poulsbo, WA; Vielight, Toronto, Canada; Bright Photomedicine, Sao Paulo, Brazil; Quantum Dynamics LLC, Cambridge, MA; Global Photon Inc, Bee Cave, TX; Medical Coherence, Boston MA; NeuroThera, Newark DE; JOOVV Inc, Minneapolis-St. Paul MN; AIRx Medical, Pleasanton CA; FIR Industries, Inc. Ramsey, NJ; UVLRx Therapeutics, Oldsmar, FL; Ultralux UV Inc, Lansing MI; Illumiheal & Petthera, Shoreline, WA; MB Lasertherapy, Houston, TX; ARRC LED, San Clemente, CA; Varuna Biomedical Corp. Incline Village, NV; Niraxx Light Therapeutics, Inc, Boston, MA. Consulting; Lexington Int, Boca Raton, FL; USHIO Corp, Japan; Merck KGaA, Darmstadt, Germany; Philips Electronics Nederland B.V. Eindhoven, Netherlands; Johnson & Johnson Inc, Philadelphia, PA; Sanofi-Aventis Deutschland GmbH, Frankfurt am Main, Germany. Stockholdings: Global Photon Inc, Bee Cave, TX; Mitonix, Newark, DE, JR, JL and JM are part of the Limburg Clinical Research Center (LCRC) and were supported by the foundation Limburg Sterk Merk, the province of Limburg, the Flemish government, Hasselt University, Ziekenhuis Oost-Limburg, Jessa Hospital, Kom op Tegen Kanker, Limburgs Kankerfonds, Limburgse Kankersamenwerking, and ASA Srl. SE discloses conflicts of interest related to treatment for cGVHD consulting Falk Pharma GmbH. PRA has been supported by travel or serves as a consultant for Mureva, Vielight, Thor Photomedicine, Kerber Applied Research, Lumithera, Jooov, and NST consulting.

The remaining authors declare that the research was conducted in the absence of any commercial or financial relationships that could be constructed as a potential conflict of interest.

## Publisher's note

All claims expressed in this article are solely those of the authors and do not necessarily represent those of their affiliated organizations, or those of the publisher, the editors and the reviewers. Any product that may be evaluated in this article, or claim that may be made by its manufacturer, is not guaranteed or endorsed by the publisher.
